# Cytosolic phospholipase A2-*α* expression in breast cancer is associated with EGFR expression and correlates with an adverse prognosis in luminal tumours

**DOI:** 10.1038/sj.bjc.6606025

**Published:** 2010-11-30

**Authors:** F Caiazza, N S McCarthy, L Young, A D K Hill, B J Harvey, W Thomas

**Affiliations:** 1 Department of Molecular Medicine, Royal College of Surgeons in Ireland, Education and Research Centre, Beaumont Hospital, Dublin 9, Ireland; 2 Department of Surgery, Royal College of Surgeons in Ireland, Education and Research Centre, Beaumont Hospital, Dublin 9, Ireland

**Keywords:** phospholipase, EGFR, breast cancer, oestrogen

## Abstract

**Background:**

The eicosanoid signalling pathway promotes the progression of malignancies through the production of proliferative prostaglandins (PGs). Cytosolic phospholipase A_2_*α* (cPLA_2_*α*) activity provides the substrate for cyclooxygenase-dependent PG release, and we have previously found that cPLA_2_*α* expression correlated with EGFR/HER2 over-expression in a small number of breast cancer cell lines.

**Methods:**

The importance of differential cPLA_2_*α* activity in clinical breast cancer was established by relating the expression of cPLA_2_*α* in tissue samples from breast cancer patients, and two microarray-based gene expression datasets to different clinicopathological and therapeutic parameters.

**Results:**

High cPLA_2_*α* mRNA expression correlated with clinical parameters of poor prognosis, which are characteristic of highly invasive tumours of the HER2-positive and basal-like subtype, including low oestrogen receptor expression and high EGFR expression. High cPLA_2_*α* expression decreased overall survival in patients with luminal cancers, and correlated with a reduced effect of tamoxifen treatment. The cPLA_2_*α* expression was an independent predictive parameter of poor response to endocrine therapy in the first 5 years of follow-up.

**Conclusion:**

This study shows a role of cPLA_2_*α* in luminal breast cancer progression, in which the enzyme could represent a novel therapeutic target and a predictive marker.

Breast cancer is the most common malignancy diagnosed in women and the leading cause of cancer-related death in women worldwide, accounting for ∼500 000 deaths per year and representing 15% of all female cancer-related mortality (Hortobagyi *et al*, 2005). Early detection and improved treatment account for the decline in mortality rates that has been observed in the past two decades (Jemal *et al*, 2007). Molecular characterisation and profiling has improved our understanding of the heterogeneity of breast cancer, and is now an important clinical tool to predict the course of the disease and identify the most appropriate treatment regimen for each patient (Perou *et al*, 2000; Sorlie *et al*, 2001; Hu *et al*, 2006). Accordingly, oestrogen receptor (ER)-positive patients are treated with adjuvant endocrine therapy (such as tamoxifen or fulvestrant; Jordan, 2007), and HER2-positive patients can benefit from targeted agents, such as trastuzumab or lapatinib (Rabindran, 2005). However, the vast majority of patients treated with adjuvant systemic therapy do not respond to the treatment or develop acquired resistance. Furthermore, patients with ER-negative, progesterone receptor (PR)-negative and HER2-negative tumours (triple-negative, or basal-like cancers) lack an established therapeutic target and can only be treated with conventional chemotherapy (Linn and Van ‘t Veer, 2009). It is, therefore, crucial to improve the translational effort in order to identify molecular markers that could aid in the prediction of tumour progression, prognosis and therapeutic regimen.

The group IVA cytosolic phospholipase A_2_ (cPLA_2_*α*) is an enzyme that catalyses the hydrolysis of the *sn*-2 linkage in membrane glycerol–phospholipids to release arachidonic acid (AA), which is then converted to biologically active eicosanoid lipid mediators, including prostaglandin E_2_ (PGE_2_), produced by cyclooxygenase (COX)-2 (Leslie, 1997). The PGE_2_ has important regulatory roles in diverse cellular responses, including cell growth and differentiation; accordingly, the AA-based eicosanoid signalling pathway has been implicated in the development and progression of cancer in different human tissues, including the breast (Nakanishi and Rosenberg, 2006; Thomas *et al*, 2008). Clinical, epidemiological and molecular evidence has linked COX-2 expression/activation and PGE_2_ production to breast cancer progression (reviewed in Howe, 2007). We have previously reported that cPLA_2_*α* can be rapidly activated by physiological concentrations of 17*β*-estradiol (E2) and is involved in the proliferative effects of E2 in breast cancer cell (Thomas *et al*, 2006, 2008; Caiazza *et al*, 2010). Specifically, the E2-induced activation of cPLA_2_*α* is mediated by *trans*-activation of EGFR/HER2 heterodimers signalling through ERK1/2 mitogen-activated protein kinase (Caiazza *et al*, 2010), resulting in the activation of proliferative signals in both ER-positive and ER-negative breast cancer cells. Pre-clinical data support a role for EGFR/HER2 signalling in promoting E2-independent tumour growth and in the development of resistance to endocrine therapy (Knowlden *et al*, 2003). In agreement with these data, clinical evidence shows that over-expression of both EGFR and HER2, which is found in 50% and 30% of breast cancers, respectively, correlates with a decreased sensitivity to endocrine therapy and with poor patient prognosis (Pietras, 2003). Furthermore, over-expression of HER2 receptors and related signalling intermediates is a molecular determinant of selective loss of ER expression, progression to ER-negative invasive phenotype and development of resistance to selective ER modulator-based therapy (Lopez-Tarruella and Schiff, 2007). Consequently, in the past 20 years, HER2 has become an important prognostic marker and therapeutic target in breast cancer. Taking into account the involvement of EGFR/HER2 receptors in the E2-induced activation of cPLA_2_*α* in breast cancer cell lines, it was hypothesised that cPLA_2_*α* activity and expression could be correlated with HER2-overexpressing tumours, a suggestion that is also supported by previous studies showing a correlation between expression of intermediates in the eicosanoid signalling pathway, particularly COX-2, and HER2 over-expression in breast cancer (Vadlamudi *et al*, 1999; Ristimaki *et al*, 2002; Subbaramaiah *et al*, 2002; Wulfing *et al*, 2003). We previously reported a novel correlation between cPLA_2_*α* and HER2 over-expression in a small number of cell lines (Caiazza *et al*, 2010). This study investigates the relationship between cPLA_2_*α* and EGFR/HER2 *in vivo*, addressing the clinical implications of differential cPLA_2_*α* expression in breast cancer.

## Patients and methods

### Patient samples and quantitative real-time PCR (qRT–PCR)

Ethical approval and patient consent was obtained for access to primary tumour samples from 18 breast cancer patients who underwent surgery at Beaumont Hospital (Dublin, Ireland) between 2008 and 2009. In all, 15 patients were ER positive and 3 were ER negative as determined by standard histopathological evaluation. The patients had not been treated with adjuvant endocrine therapy; however, the majority had received selective oestrogen receptor modulator therapy with tamoxifen. The complete clinicopathological characteristics are summarised in Supplementary Table S1.

RNA extraction and qRT–PCR were performed as previously described (Caiazza *et al*, 2010). Expression levels of HER2 mRNA were normalised to a standard housekeeping gene (*18S rRNA*) and calibrated to the MCF-7 cell line chosen to represent 1 × expression of *HER2* gene (Barberis *et al*, 2008) All samples above a threshold of HER2 mRNA expression relative to the calibrator were assigned to the HER2-positive group. This method was previously validated by comparing HER2 mRNA levels in 55 formalin-fixed, paraffin-embedded samples, in which HER2 status was evaluated by IHC and fluorescence *in situ* hybridisation (Barberis *et al*, 2008).

### Gene expression microarrays

The expression of PLA2G4A (probe 210145_at), EGFR (probe 210984_x_at) and ERBB2 (probe 210930_s_at) was examined in two previously published microarray data sets of breast cancer cell lines (Kao *et al*, 2009) and primary tumour samples (van de Vijver *et al*, 2002), which had been profiled with an Affymetrix microarray assay (Affymetrix, Santa Clara, CA, USA). Data were downloaded, respectively, from the PLOS website (http://dx.plos.org/10.1371/journal.pone.0006146) and from the original publication on-line supporting information website (http://microarray-pubs.stanford.edu/ wound_NKI/explore.html). Comprehensive clinical data of all 295 patients were also available from the same source.

### Statistical analysis

Statistical analysis of the data was performed with paired Student's *t*-test for analysis between two groups. Fisher's exact test was performed on contingency tables to analyse the correlation between two variables. Pearson's coefficient was used to measure correlation between linear variables. Survival curves were calculated according to the Kaplan–Meier method and compared using the Cox–Mantel log-rank test. Overall survival (OS) was calculated as the time between the first date of treatment and the last date of follow-up or the date of death. Relapse-free survival (RFS) was calculated as the time between the first date of treatment and the last date of follow-up or date of recurrence (loco-regional recurrence or distant metastasis). Kaplan–Meier curves were calculated, as indicated, over the entire follow-up time (17 years) or over a follow-up time of 10 years to focus on the effects on early development of acquired endocrine resistance. All analyses were performed using prism (Graphpad Software, La Jolla, CA, USA). Cox proportional hazard models were computed using XLStat-Life for Microsoft Excel (Kovach Computing Services, Anglesey Wales). The cPLA_2_*α* expression was categorised as high or low using the upper median as a cutoff point. Where indicated, the median was used as a cutoff point to exclude any possible bias because of the low number of patients. All other variables were either dichotomised or analysed as continuous variables as indicated. The number of patients in each group is reported in the figure legend. A heat-map image was generated from microarray data using the Matrix2png web interface (University of British Columbia, http://chibi.ubc.ca/matrix2png; Pavlidis and Noble, 2003). All *P*-values are two-tailed, and *P*-values of <0.05 were considered statistically significant.

## Results

### Increased expression of cPLA_2_*α* mRNA correlates with HER2 over-expression in breast cancer patients

**Figure 1:**
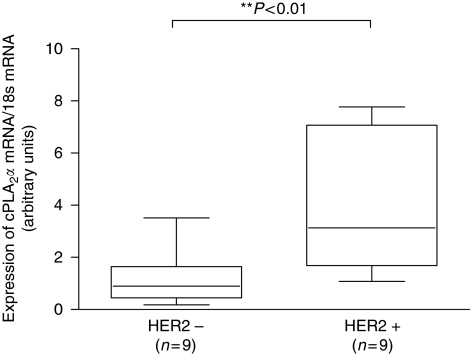
Expression of cPLA_2_*α* mRNA according to HER2 expression in breast cancer tissue samples. Samples from 18 breast cancer patients were separated into HER2-positive and HER2-negative groups according to qRT–PCR quantification and threshold of HER2 expression, as indicated in Materials and methods. Expression of cPLA_2_*α* mRNA, measured by qRT–PCR, was compared amongst the two groups. ^**^P<0.01 measured by Student's *t*-test.

The expression of cPLA_2_*α* was analysed by qRT–PCR in tumour samples from a cohort of Irish breast cancer patients (complete clinical data for the 18 patients are detailed in Supplementary Table S1). Recently, the validity of IHC to test for HER2 amplification has been questioned in terms of standardisation, reproducibility and accuracy (Gown, 2008), and different groups have reported results supporting a better accuracy of qRT–PCR in detecting HER2 amplification, avoiding false positives and false negatives (Vinatzer *et al*, 2005; Kulka *et al*, 2006). Consequently, we quantified HER2 expression in all 18 samples using qRT–PCR. In all, 9 samples out of 18 (50%) scored as HER2 positive using the described method, including some that were previously classified as HER2 negative using standard pathological evaluation. Expression of cPLA_2_*α* mRNA was significantly (*P*<0.01) increased in HER2-positive tissue samples (Figure 1).

### Increased expression of cPLA_2_*α* mRNA correlates with the basal-like and HER2-positive subtypes, and with markers of poor prognosis in a panel of breast cancer cell lines

**Figure 2:**
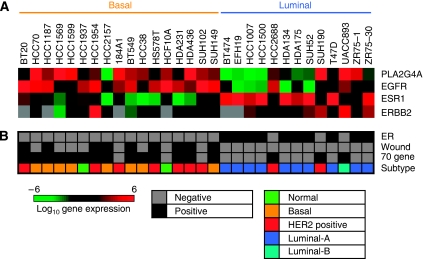
Correlation between cPLA_2_*α* mRNA expression and clinicopathological characteristics in a panel of 30 breast cancer cell lines. (**A**) Log_2_ expression levels of cPLA_2_*α* (PLA2G4A), ER*α* (ESR1), EGFR and HER2 (ERBB2) are depicted by the indicated pseudo-colour scale (grey represents missing or poorly measured data). (**B**) Classification of cell lines by ER status, by positivity for 70 gene and wound response signatures, and by nearest resemblance to tumour gene expression subtype (normal, luminal A, luminal B, HER2 positive, basal-like).

With the aim of expanding our study to a larger sample size population, we first analysed the expression of cPLA_2_*α* in a panel of 30 breast cancer cell lines for which gene expression microarray data were previously published (Kao *et al*, 2009). Increased expression of cPLA_2_*α* correlated with high expression of EGFR and with low expression of ER*α* at the mRNA level (Figure 2A). No significant correlation was found between cPLA_2_*α* and HER2 expression, but this was influenced by missing data for HER2 mRNA expression levels. High cPLA_2_*α* abundance clustered in cell lines characterised by basal phenotype compared with luminal phenotype, and cPLA2*α* expression also correlated with basal-like and HER2-positive subtypes as opposed to luminal A and luminal B subtypes (Figure 2B). High EGFR expression, low ER*α*, the basal and HER2-positive subtypes are all molecular markers of poor prognosis in breast cancer. High cPLA_2_*α* expression also correlated with two different profile signatures that are predictive of poor prognosis: the 70 gene (van ‘t Veer *et al*, 2002) and the wound response (Chang *et al*, 2005) signatures (Figure 2B).

### Increased expression of cPLA_2_*α* mRNA correlates with clinical parameters of poor prognosis in a cohort of breast cancer patients

**Figure 3:**
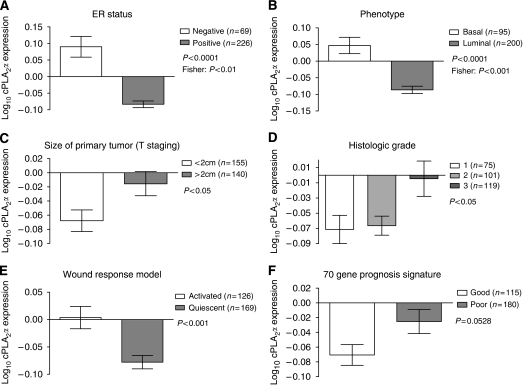
Correlation between cPLA_2_*α* mRNA expression and clinicopathological characteristics in 295 breast cancer patients. Log_10_ expression of cPLA_2_*α* compared with (**A**) ER status, (**B**) basal-luminal phenotype, (**C**) size of primary tumour, (**D**) histology grade, (**E**) the wound response signature and (**F**) the 70 gene signature. The number of patients (*n*) in each group is indicated. *P*-values were calculated with Student's t-test, or with Fisher's exact test (where indicated).

**Figure 4:**
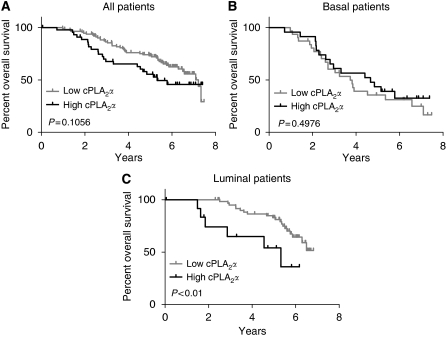
Prognostic value of cPLA_2_*α* expression on the overall survival of 295 breast cancer patients. Kaplan–Meier analysis of the probability of overall survival among (**A**) all 295 breast cancer patients (high *n*=45 *vs* low *n*=112), or patients with (**B**) basal (high *n*=23 *vs* low *n*=31) and (**C**) luminal cancers (high *n*=13 *vs* low *n*=62), according to cPLA_2_*α* expression. Survival curves were compared with the Cox-Mantel log-rank test, and the corresponding *P*-value is indicated.

Expression of cPLA_2_*α* was investigated in a second gene expression microarray dataset comprising 295 breast cancer patients from The Netherland Cancer Institute (van de Vijver *et al*, 2002). There was a positive correlation between cPLA_2_*α* and EGFR mRNA expression (Pearson *R*^2^=0.056, *P*<0.0001), but no correlation between cPLA_2_*α* and HER2 expression (*P*=0.7816). High cPLA_2_*α* mRNA abundance correlated with ER-negative status as opposed to ER positive (*P*<0.0001), and with basal phenotype compared with luminal (*P*<0.0001; Figure 3A and B and Supplementary Table S2). When compared with the TNM staging system, high cPLA_2_*α* expression correlated with size of the primary tumour (T) of >2 cm (*P*<0.05; Figure 3C) but not with lymph node involvement (N) or with distant metastasis (M). Increased cPLA_2_*α* abundance also correlated with histology grade 3 tumours compared with grade 1 and 2 (*P*<0.05) and with the activated wound response model (a gene signature predictive of poor prognosis (Chang *et al*, 2005)) (*P*<0.001). Correlation with the 70 gene profile signature almost reached statistical significance (*P*=0.0528; Figure 3D–F and Supplementary table S2). In spite of the correlation between high cPLA_2_*α* mRNA expression and different clinical parameters characteristic of poor prognosis, cPLA_2_*α* itself was not associated with poor prognosis in both univariate and multivariate analysis (Figure 4 and Supplementary Table S3). Patients with high cPLA_2_*α* expression had a reduced 7 years OS compared with patients expressing low cPLA_2_*α*, but this was not statistically significant (*P*=0.1; Figure 4A). However, when the same analysis was repeated separately on luminal and basal tumour patient data, high cPLA_2_*α* expression was associated with reduced OS in patients with luminal cancer (*P*<0.01; Figure 4B and C).

### Increased expression of cPLA_2_*α* correlates with poor response to endocrine therapy in breast cancer patients

**Figure 5:**
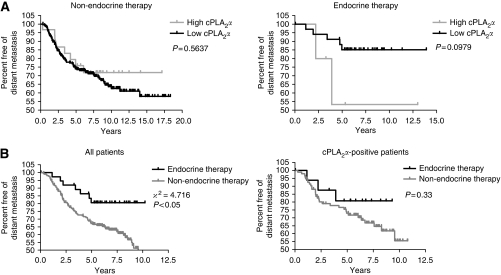
Role of cPLA_2_*α* in the early development of endocrine resistance in 295 breast cancer patients. Kaplan–Meier analysis of the probability that patients would remain free of distant metastases (**A**) among patients treated only with radiotherapy/chemotherapy and patients treated with adjuvant tamoxifen therapy, according to cPLA_2_*α* expression (high, *n*=31 *vs* low, *n*=224 for radio/chemotherapy and high, *n*=5 *vs* low, *n*=35 for adjuvant therapy), total follow-up time of 17 years; (**B**) among all patients or patients with high cPLA_2_*α* expression, according to the presence or absence of adjuvant endocrine therapy (non-endocrine, *n*=203 *vs* endocrine, *n*=38 for all patients; non-endocrine, *n*=94 *vs* endocrine, *n*=17 for cPLA_2_*α* positive patients), follow-up time of 10 years; (**A** and **B**) Survival curves were compared using the Cox–Mantel log-rank test. The corresponding *χ*^2^ values and *P*-values are indicated. The threshold for cPLA_2_*α* dichotomisation in (**B**) was lowered to the median in order to include more patients; the statistical significance was retained as compared with the analysis with the median cutoff point (not shown), suggesting the absence of bias because of the low number of patients.

Increased expression of EGFR/HER2 and decreased expression of ER*α* are molecular markers associated with resistance to endocrine therapy in breast cancer (Pietras, 2003). We investigated the relationship between cPLA_2_*α* expression and the development of endocrine resistance in The Netherland Cancer Institute cohort of 295 breast cancer patients (see Supplementary Table S4 for subset sizes). Although patients who expressed cPLA_2_*α* most highly, had a reduced RFS when treated with endocrine therapy compared with patients who received no adjuvant hormonal treatment, according to Kaplan–Meier univariate survival analysis, these data did not reach statistical significance (*P*=0.56 and *P*=0.09, respectively; Figure 5A). However, the increased RFS measured in patients treated with adjuvant hormonal therapy compared with patients only treated with local therapy (log-rank test, *P*<0.05) was completely lost in the subset of patients expressing high levels of cPLA_2_*α* (*P*=0.33), with the most pronounced difference between the cPLA_2_*α*-positive patients and the whole cohort observed within the first 5 years after diagnosis (Figure 5B). For the whole cohort of patients, endocrine therapy increased the mean time before first metastasis by 1 year compared with receiving local therapy only (8.52±0.45 and 7.54±0.29 years, respectively), and increased the number of patients free of distant metastasis 5 years after diagnosis by 18% (76.3% with hormonal therapy *vs* 58.6% with no hormonal therapy). These effects were lost in high cPLA_2_*α*-expressing patients, for both the mean time before first metastasis (7.11±0.72 years with hormonal therapy *vs* 7.96±0.42 years without hormonal therapy) and the percentage of patients free of distant metastasis at 5 years after diagnosis (64.7% with hormonal therapy *vs* 64.9% without hormonal therapy). A Cox proportional multivariate analysis performed on this data set (Table 1) showed that low cPLA_2_*α* mRNA expression and adjuvant hormonal therapy were independent prognostic factors, and were predictive of increased RFS in the first 5 years of patient follow-up (odds ratio: 0.244, 95% CI: 0.069–0.862, *P*<0.05). This analysis also included ER and HER2 expression as continuous variables.

## Discussion

The role of eicosanoid signalling in carcinogenesis has been the subject of intense investigation over the past 20 years, particularly in the context of COX-2 over-expression. The dysregulation of cPLA_2_*α*, through alterations in its functional activity or in expression levels, is also a common feature of many types of human cancer including mammary adenocarcinoma, leading to high levels of proliferative eicosanoids (Nakanishi and Rosenberg, 2006; Thomas *et al*, 2008). Antagonism of cPLA_2_*α* would limit free intracellular arachidonic acid availability; this in turn would have a dual effect on tumour cells by reducing proliferative PGE_2_ production and also block the supply of arachidonic acid to alternate pathways, such as those mediated by COX-1 and lipoxygenase. This alternate metabolism of arachidonic acid represents a serious negative side effect that was reported with the clinical use of COX-2-specific inhibitors, which may be over come through specific cPLA_2_*α* antagonism (Laye and Gill, 2003). We previously demonstrated that selective pharmacological inhibition of cPLA_2_*α* leads to the growth inhibition of breast cancer cells *in vitro* (Caiazza *et al*, 2010). It is, therefore, crucial to determine whether cPLA_2_*α* has any value as a putative therapeutic target *in vivo*, and also to investigate which specific subset of breast cancer patients may benefit the most from such intervention. We previously found through *in vitro* studies that there was a correlation between high cPLA_2_*α* expression and HER2 over-expression at both mRNA and protein level in a small panel of breast cancer cell lines (Caiazza *et al*, 2010). This expression correlation may be explained by a bi-directional cross-talk between the two proteins; where by HER2 can regulate the expression of cPLA_2_*α* which in turn functions by modulating the transcription of HER2 through the production of PGE_2_ (Benoit *et al*, 2004; Caiazza *et al*, 2010). A similar synergy was also reported between HER2 and COX-2 expression (Benoit *et al*, 2004; Wang *et al*, 2004), and as a consequence COX-2 over-expression is associated with HER2-driven tumourigenesis *in vivo* (Howe *et al*, 2005; Dillon *et al*, 2008). In this study, we report a correlation between cPLA_2_*α* and high HER2 mRNA expression in a group of 18 breast cancer patients. This was subsequently confirmed by the analysis of a gene expression microarray of 30 breast cancer cell lines, in which high mRNA expression of cPLA_2_*α* correlated with high EGFR expression and with the HER2-positive and basal-like subtypes of breast cancer in cell lines showing basal phenotype. The molecular classification of breast cancer, based on the seminal work of Perou *et al*, 2000 (Sorlie *et al*, 2001), identified five main subtypes of tumours with distinct patterns of gene expression and different disease outcomes: luminal A and luminal B cancers (mainly ER positive and sensitive to endocrine therapy), HER2 positive (ER negative or with low ER expression, HER2 over-expressing and resistant to endocrine therapy), basal-like (ER negative, PR negative and HER2 negative, also called triple negative) and normal-like. In our *in silico* analysis of gene expression microarrays from both cell lines and patients, elevated cPLA_2_*α* mRNA expression was associated with clinical parameters for poor prognosis, which correlate with the HER2-positive and basal-like subtypes of breast cancer. Low ER expression, high EGFR expression, activation of poor prognosis profile signatures, large tumour size and high histological grade all associated significantly with high cPLA_2_*α* mRNA expression. So supporting the hypothesis that cPLA_2_*α* could represent a specific therapeutic target for a clinically challenging subset of breast cancer patients with highly invasive, endocrine resistant tumours of the HER2-positive or triple-negative subtype. In spite of the association between cPLA_2_*α* expression and different clinicopathological parameters characteristic of poor prognosis, cPLA_2_*α* was not directly associated with poor prognosis, but only correlated with reduced overall survival in patients with the luminal type of cancer. This result suggested that cPLA_2_*α* could predict prognosis specifically in luminal breast cancers, and hence be indicative of the response to endocrine therapy. Over-expression of EGFR/HER2 and downregulation of ER are molecular markers associated with *de novo* and acquired resistance to endocrine therapy in both pre-clinical models of breast cancer and in clinical studies (Knowlden *et al*, 2003; Schiff *et al*, 2005). Several studies have provided indirect evidence of a role for the eicosanoid signalling pathway in the development of endocrine resistance, including the PGE_2_-mediated, enhanced expression of HER2 (Benoit *et al*, 2004) and expression of aromatase (Zhao *et al*, 1996). Direct evidence also supports a role for COX-2 as an independent prognostic marker for poor response to tamoxifen treatment in breast cancer patients (Dillon *et al*, 2008). In this study, we analysed cPLA_2_*α* expression with regards to hormonal treatment in a population of 295 breast cancer patients that were either treated with adjuvant tamoxifen (alone or in combination with chemotherapy) or did not receive any adjuvant endocrine treatment. High cPLA_2_*α* mRNA was measured in only a small subset of the patients who received endocrine therapy, and increased cPLA_2_*α* expression in this group predicted poor response to endocrine therapy in the first 5 years of follow-up in univariate analysis. The cPLA_2_*α* was also an independent prognostic marker of poor response to endocrine therapy in a multivariate analysis that also included hormonal treatment, ER and HER2 expression to adjust for any bias because of the association of cPLA_2_*α* expression with ER negativity (which itself correlates with endocrine resistance). This analysis only takes into account treatment with tamoxifen and does not include other types of endocrine therapies, such as aromatase inhibition.

In summary, the data presented in this work provide evidence of a role for cPLA_2_*α* in breast cancer progression; cPLA_2_*α* expression correlated with clinicopathological parameters of poor prognosis and with highly aggressive tumours characterised by low ER expression, high EGFR expression and basal phenotype. This work also provides evidence to support a role for cPLA_2_*α* as a predictive marker for poor prognosis, and suggest a role in the early onset of resistance to tamoxifen therapy, in luminal cancers; this is in common with what has been previously reported for COX-2 expression (Dillon *et al*, 2008). Targeting eicosanoid pathway intermediates may prove a valuable strategy to overcome acquired resistance to tamoxifen treatment of breast cancer, and it has been suggested that specific antagonism of cPLA_2_*α* could be useful in augmenting treatment (Thomas *et al*, 2008). In this study, we show that patients with highly aggressive cancers of the HER2-positive and basal-like subtypes, as well as patients with luminal tumours who developed resistance to endocrine therapy, are most likely to benefit from such a therapeutic strategy, due to their higher cPLA_2_*α* expression levels.

## Figures and Tables

**Table 1:** Multivariable proportional-hazard (cox) analysis of the risk of distant metastasis as a first event within 5 years of diagnosis

**Variable**	**Hazard ratio (95% CI^a^)**	***P*-value**
Low cPLA_2_*α* (*vs* high)	0.244 (0.069–0.862)	0.028
Hormonal therapy (*vs* no hormonal therapy)	0.384 (0.172–0.855)	0.019

Abbreviations: CI=confidence interval, cPLA_2_*α*=cytosolic phospholipase A_2_*α*; ER=oestrogen receptor.

a CI (adjusted for ER and HER2 status).

Total patients=124 (hormonal=20, no hormonal=104, PLA2*α* high=4, PLA2*α* low=120).
