# Supplementary material for: Cytosolic phospholipase A2-α expression in breast cancer is associated with EGFR expression and correlates with an adverse prognosis in luminal tumours
Source: Br J Cancer. 2010 Nov 30;104(2):338–44. doi: 10.1038/sj.bjc.6606025 (PMC3031888; doi:10.1038/sj.bjc.6606025)
Supplement: Supplementary Table S1 [file 6606025x1.pdf]

Table S1: Clinical characteristics of the Irish patients cohort

Variable	no. of patients	%
Total patients	18	100
Age mean	55 (years)	
range	33-77 (years)	
ER status		
positive	15	83
negative	3	17
PR status		
positive	7	39
negative	11	61
HER2 status		
positive	4	22
negative	14	78
Tumor type		
IDC	15	83
ILC	2	11
DCIS	1	6
Tumor grade		
1	1	6
2	8	44
3	9	50

ER (Estrogen Receptor), PR (Progesteron Receptor), IDC (Invasive Ductal Carcinoma), ILC (Invasive Lobular Carcinoma), DCIS (Ductal Carcinoma In Situ)
