# Supplementary material for: Cytosolic phospholipase A2-α expression in breast cancer is associated with EGFR expression and correlates with an adverse prognosis in luminal tumours
Source: Br J Cancer. 2010 Nov 30;104(2):338–44. doi: 10.1038/sj.bjc.6606025 (PMC3031888; doi:10.1038/sj.bjc.6606025)
Supplement: Supplementary Table S2 [file 6606025x2.pdf]

Table S2: Association between clinical characteristics and expression levels of $cPLA_2$

Characteristic	mean $\log_{10}$ $cPLA_2$ expression (±SEM)	P value
Estrogen Receptor status		**<0.0001**
negative (n=69)	0.0900 (± 0.03110)	
positive (n=226)	-0.08303 (± 0.01011)	
Phenotype		**<0.0001**
luminal (n=200)	-0.08518 (± 0.01098)	
basal (n=95)	0.04718 (± 0.02456)	
Size of primary tumor (T staging)		**<0.05**
<2 cm. (n=155)	-0.06735 (± 0.01533)	
>2 cm. (n=140)	-0.01511 (± 0.01682)	
Lymph nodes involvement (N staging)		0,199
no (n=151)	-0.02821 (± 0.01849)	
yes (n=144)	-0.05760 (± 0.01306)	
Metastases (M staging)		0,9373
no (n=194)	-0.04321 (± 0.01473)	
yes (n=101)	-0.04131 (± 0.01777)	
Tumor grade		**<0.05**
1 (n=75)	-0.07121 (± 0.01860)	
2 (n=101)	-0.06618 (± 0.01254)	
3 (n=119)	-0.004445 (± 0.02314)	
70 genes prognosis signature		0,0528
good prognosis (n=115)	-0.07021 (± 0.01406)	
poor prognosis (n=180)	-0.02489 (± 0.01632)	
Wound response model		**<0.0001**
activated (n=126)	0.003730 (± 0.02042)	
quiescent (n=169)	-0.07707 (± 0.01227)	

P values were calculated with Student's t test.
Significant values are highlithed in bold.
