# Supplementary material for: Cytosolic phospholipase A2-α expression in breast cancer is associated with EGFR expression and correlates with an adverse prognosis in luminal tumours
Source: Br J Cancer. 2010 Nov 30;104(2):338–44. doi: 10.1038/sj.bjc.6606025 (PMC3031888; doi:10.1038/sj.bjc.6606025)
Supplement: Supplementary Table S3 [file 6606025x3.pdf]

Table S3: Multivariable proportional-hazard (Cox) analysis of the risk of death or the risk of developing a metastasis as a first event

Variable	Overall Survival Hazard Ratio (95% CI*)	Overall Survival P value	Relapse-Free Survival Hazard Ratio (95% CI*)	Relapse-Free Survival P value
High $cPLA_2$ (vs. low)	2.4 (0.8-7.0)	0,11	1.1 (0.4-3.2)	0,8
Tumor size <2cm (vs. >2cm)	0.48 (0.3-0.77)	**<0.01**	0.4 (0.26-0.64)	**<0.0001**
Tumor grade				
grade 2 (vs. grade 1)	4.6 (1.6-13.3)	**<0.01**	2.7 (1.3-5.7)	**<0.01**
grade 3 (vs. grade 1)	10 (3.6-27.7)	**<0.0001**	4.6 (2.3-9.4)	**<0.0001**
Tumor diameter (mm.)	1.04 (1.01-1.06)	**<0.01**	1.05 (1.02-1.07)	**<0.0001**
ER positive (vs. ER negative)	0.3 (0.18-0.48)	**<0.0001**	0.46 (0.28-0.74)	**<0.01**
Lymph node involvement (vs. no involvement)	1.07 (0.98-1.17)	0,1	1.09 (1.0-1.18)	0,036
Age (years)	0.95 (0.91-0.98)	**<0.01**	0.94 (0.91-0.98)	**<0.01**
No chemotherapy (vs. chemotherapy)	1.28 (0.8-2)	0,3	1.46 (0.93-1.93)	0,1
No hormonal therapy (vs. hormonal therapy)	1.62 (0.7-3.7)	0,25	1.79 (0.82-3.88)	0,14

* Confidence Interval

Analysed as a continuous variable
