# Supplementary material for: Cytosolic phospholipase A2-α expression in breast cancer is associated with EGFR expression and correlates with an adverse prognosis in luminal tumours
Source: Br J Cancer. 2010 Nov 30;104(2):338–44. doi: 10.1038/sj.bjc.6606025 (PMC3031888; doi:10.1038/sj.bjc.6606025)
Supplement: Supplementary Table S4 [file 6606025x4.doc]

| **Table S4:** Subset sizes for Figure 5 analysis | | |
| --- | --- | --- |
|  |  | **n** |
| Non-endocrine | High cPLA_2_ | 31 |
|  | Low cPLA_2_ | 224 |
|  |  |  |
| Endocrine | High cPLA_2_ | 5 |
|  | Low cPLA_2_ | 35 |
|  |  |  |
| All patients | Non-endocrine | 203 |
|  | Endocrine | 38 |
|  |  |  |
| cPLA_2_ positive | Non-endocrine | 94 |
|  | Endocrine | 17 |
|  |  |  |
| All ER positive | Non-endocrine | 166 |
|  | endocrine | 28 |
|  |  |  |
| ER positive high cPLA_2_ | Non-endocrine | 26 |
|  | endocrine | 7 |
